# Silibinin Causes Apoptosis and Cell Cycle Arrest in Some Human Pancreatic Cancer Cells

**DOI:** 10.3390/ijms12084861

**Published:** 2011-08-02

**Authors:** Yakun Ge, Yuanxin Zhang, Yunpeng Chen, Quanshun Li, Jun Chen, Ying Dong, Wei Shi

**Affiliations:** 1 Key Laboratory for Molecular Enzymology & Engineering of the Ministry of Education, School of Life Science, Jilin University, Changchun 130023, China; E-Mails: yakunge@126.com (Y.G.); zhangyuanxin@126.com (Y.Z.); qsli06@mails.jlu.edu.cn (Q.L.); 2 College of Environmental & Biological Engineering, Jilin Institute of Chemical Technology, Jilin 132022, China; 3 The Center for Combinatorial Chemistry and Drug Discovery, the College of Pharmacy, Jilin University, Changchun 130021, China; E-Mail: cyp830215@163.com; 4 School of Pharmacy, Jiangsu University, Zhenjiang, Jiangsu 212013, China; E-Mails: shchen@ujs.edu.cn (J.C.); ydong@ujs.edu.cn (Y.D.)

**Keywords:** Silibinin, cell proliferation, apoptosis, cell-cycle arrest, pancreatic cancer

## Abstract

Silibinin, an effective anti-cancer and chemopreventive agent in various epithelial cancer models, has been reported to inhibit cancer cell growth through mitogenic signaling pathways. However, whether it can inhibit human pancreatic carcinoma growth and what are the underlying mechanisms is still not well elucidated. Here, we evaluated the inhibitory proliferation effects of Silibinin in pancreatic carcinoma growth and examined whether Silibinin modulates cell cycle and apoptosis. Our results indicate that Silibinin effectively inhibited the pancreatic carcinoma AsPC-1, BxPC-3 and Panc-1 cells’ proliferation and caused apoptosis. Silibinin induced a decrease in S phase and cell cycle arrest in G1 phase in AsPC-1 cells, but had no obvious changes in BxPC-3 and Panc-1 cell cycle. Furthermore, these results suggest that Silibinin might be a candidate chemopreventive agent for pancreatic carcinoma therapy.

## 1. Introduction

Silibinin is a major active constituent of silymarin, a mixture of flavonolignans extracted from milk thistle (*Silybum marianum*) [1]. Milk thistle extract has centuries-old history of use in folk medicine to treat a variety of illnesses including jaundice, gallstones, hemorrhage, bronchitis, varicose vein, and for several other purposes [2]. Silibinin is well tolerated and largely free of any adverse effects. Silibinin is nontoxic in acute, subchronic and chronic tests even at large doses, and there is no known LD_50_ for Silibinin in animal studies [3–6]. The anticancer efficacy of Silibinin is clearly evident from the published reports against various cancers including prostate, skin, lung, colon, breast, hepatic, ovarian, cervical, kidney, gastric carcinoma [2], and the underlying mechanisms are very different in different cancer cells. For instance, Silibinin activates p53 and induces autophagic death in human fibrosarcoma HT1080 cells via reactive oxygen species-p38 and c-Jun N-terminal kinase pathways [1]. Silibinin induces apoptosis by down-regulating survivin through inhibition of hypoxia-inducible factor-1 alpha in non-small cell lung cancer cells [7]. Silibinin activates p53-caspase 2 pathways and causes caspase-mediated cleavage of Cip1/p21 in apoptosis induction in bladder transitional-cell papilloma RT4 cells [8]. Silibinin up-regulates the expression of cyclin-dependent kinase inhibitors and causes cell cycle arrest and apoptosis independent of caspases activation in human colon carcinoma HT-29 cells [9].

Pancreatic cancer is a malignant neoplasm of the pancreas. It is estimated that in 2010 more than 43,000 individuals in the United States have been diagnosed with this condition, and 36,800 have died from this disease [10]. The oncogenesis of pancreatic cancer in particular appears to favor the development and subsequent expansion of cell clones that are resistant to apoptotic triggers [11]. Therefore, an important research objective is the identification of lead compounds that circumvent the resistance mechanisms that limit the success of conventional drugs. Anticancer activities of Silibinin in pancreatic cancer have not been examined and the underlying mechanism has not been clarified yet.

The purpose of this work was to develop an understanding of Silibinin’s effects on pancreatic cancer cells to begin to determine its therapeutic value in preventing or treating this disease. Therefore we examined the anticancer activities of Silibinin, and investigated its mechanism in three pancreatic cancer cell lines, AsPC-1, BxPC-3 and Panc-1. Silibinin activated caspase 3, 8, 9 and caused cell apoptosis. Silibinin induced a decrease in S phase and cell cycle arrest in G1 phase in AsPC-1 cells, but resulted in no obvious changes in BxPC-3 and Panc-1 cell cycles.

## 2. Results and Discussion

### 2.1. Effect of Silibinin on AsPC-1, Panc-1 and BxPC-3 Cell Growth Inhibition

**Figure 1.**
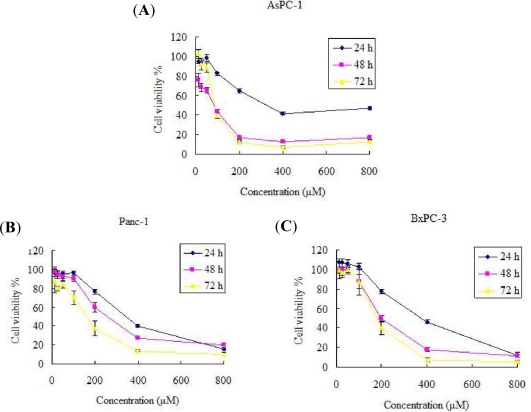
Silibinin inhibits proliferation of AsPC-1 (**A**), Panc-1 (**B**) and BxPC-3 (**C**) cells. Cells were plated at a density of 5000 cells per well and then treated with 2‰ (v/v) DMSO (Control) or with different doses of Silibinin (12.5, 25, 50, 100, 200, 400 and 800 μM) for 24, 48 and 72 h. Cell viability was then determined by a MTT assay. Significance was determined by a Student’s t-test (*p* < 0.05, compared with control).

To examine the effects of Silibinin on AsPC-1, Panc-1 and BxPC-3 cancer cell growth, we conducted a dose escalation experiment (Figure 1). The results indicate that Silibinin inhibited the proliferation of AsPC-1, Panc-1 and BxPC-3 cells in a dose- and time-dependent manner. Dimethyl sulfoxide (DMSO) is primarily used as a solvent for pharmaceutics and as a cryopreservant of cell lines. In this text, no differences were found between 2‰ (v/v) DMSO and the negative control. There was a significant decrease in cell proliferation in Panc-1 and Bxpc-1 cells observed after 48 h treatment with 200 μM Silibinin. However, for AsPC-1 cells, there was a significant decrease in cell proliferation at a concentration of 100 μM Silibinin. That is, different cancer cells, even if coming from the same tissue, have different sensitivities to Silibinin.

### 2.2. Effect of Silibinin on the Induction of Apoptosis

**Figure 2.**
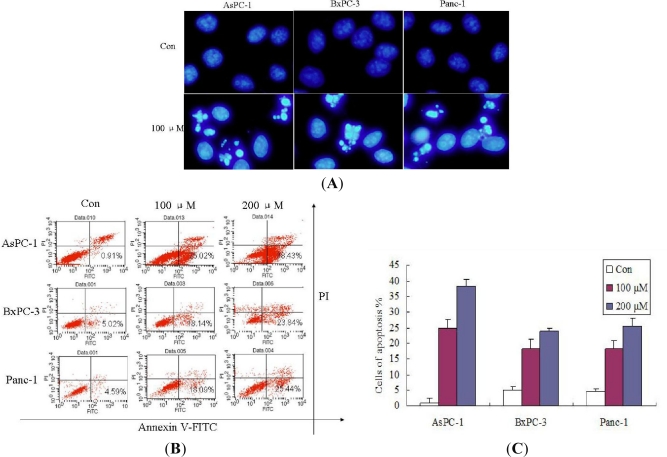
Silibinin induced cell apoptosis in AsPC-1, Panc-1 and BxPC-3 cells. (**A**) Cells were treated with 2‰ (v/v) DMSO (Control) and 200 μM Silibinin for 24 h. Cells were then fixed and stained with DAPI. Stained nuclei were observed under a fluorescent microscope using a blue filter; (**B**) Cells treated with 100, 200 μM of Silibinin for 48 h were assessed for apoptosis by staining with Annexin V-FITC and PI; (**C**) Cells treated with 100, 200 μM of Silibinin for 48 h were assessed for apoptosis by staining with Annexin V-FITC and PI. The results are shown from one of three experiments with similar results. Each point represents the mean ± SD of three independent experiments. Significance was determined using a Student’s t-test (*p* < 0.05, compared with control).

The growth inhibitory effects of Silibinin were evaluated to determine if they correlated with the induction of apoptosis. Cancer develops when the balance between cell proliferation and cell death is disturbed, and the aberrant cell proliferation leads to tumor growth. It is well known that apoptosis and its related signaling pathways have a profound effect on the progression of cancer [12], suggesting that agents inducing apoptotic death of human cancer cells, including pancreatic cancer, may play a critical role in cancer prevention/intervention. Morphological analysis using DAPI staining revealed the presence of nuclei with chromatin condensation and the formation of apoptotic bodies in cells cultured with Silibinin. In this study, no differences were found between 2‰ (v/v) DMSO and negative control (Figure 2A). In addition, flow cytometric analyses revealed enhanced apoptosis of AsPC-1, Panc-1 and BxPC-3 cells treated with Silibinin in a concentration dependent manner (Figure 2B and 2C), based on the formation of a significant accumulation of early apoptosis (Annexin V-positive and PI-negative) cells. In AsPC-1 cells, after 100 μM Silibinin treatment for 24, 48, and 72 h, percentages of apoptotic cells were 13.24%, 25.02%, and 29.03%. In BxPC-3, after 100 μM Silibinin treatment for 24, 48, and 72 h, percentages of apoptotic cells were 7.02%, 18.14%, and 23.03%. In Panc-1, after 100 μM Silibinin treatment for 24, 48, and 72 h, percentages of apoptotic cells were 6.03%, 15.09%, and 20.34%. These results revealed enhanced apoptosis of AsPC-1, Panc-1 and BxPC-3 cells treated with Silibinin in a time dependent manner. All these findings demonstrated that the inhibition effects observed in response to Silibinin were mainly associated with the induction of apoptotic cell death in AsPC-1, Panc-1 and BxPC-3 cells. However, AsPC-1 was the most sensitive cell line to Silibinin. Several classes of chemotherapy drugs cause apoptotic death of cancer cells, but their non-selective efficacy (toxicity) in other tissues has been a limitation in their efficacy. Our data demonstrating significant apoptotic death induction by Silibinin in AsPC-1 cells, but only slightly in Panc-1 and BxPC-3 cells, suggested the possibility of both selectivity and specificity in Silibinin efficacy against pancreatic AsPC-1 cells.

### 2.3. Silibinin Triggers Apoptosis by Activating the Caspases

It is well known that chemotherapeutic agents induce apoptosis in most cells via two major pathways: death receptor-mediated pathway and mitochondria-mediated pathway [13,14]. Both pathways converge to a final common pathway involving the activation of a cascade of proteases called caspases, which can cleave regulatory and structural molecules, and thus induce cell death [15]. Silibinin induced cell apoptosis both in a manner dependent on caspases activation [8,16] or independent of caspases activation [9] in human carcinoma cells.

**Figure 3.**
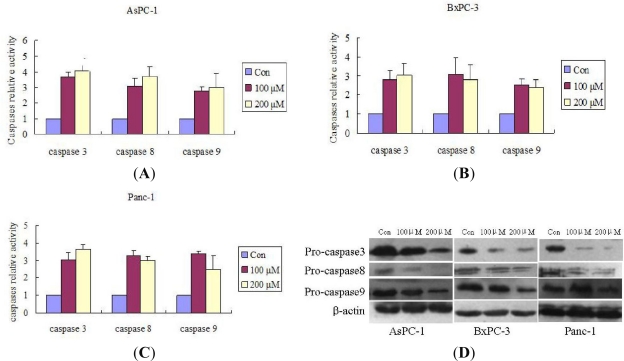
Silibinin triggers apoptosis by activating caspases. (**A**–**C**) Cells were incubated with 100 or 200 μM Silibinin for 48 h. Equal amounts of cell lysates were assayed for caspase-3, caspase-8 and caspase-9 activity using DEVD-pNA, IETD-pNA and LEHD-pNA as substrates, respectively. The concentrations of the fluorescent products released were then measured; (**D**) Cells were incubated with 0, 100, 200 μM of Silibinin for 24 h. Equal amounts of cell lysates were then resolved by SDS-PAGE, transferred to PVDF membrane, and probed with anti-caspase 3, 8, or 9 antibodies. The proteins were then visualized using a detection system. β-actin was used as an internal control. Results represent the mean ± SD of triplicate determinations. The significance was determined by a Student’s t-test (*p* < 0.05, compared with control).

We first investigated the effect of Silibinin on caspase-3 (an effector caspase). The results show the caspases involvement in Silibinin-triggered apoptosis in AsPC-1, Panc-1 and BxPC-3 cells. Silibinin significantly cleaved pro-caspase-3 and activated the caspase-3. The effect of Silibinin on caspase-8 and caspase-9 were then evaluated, and Silibinin cleaved pro-caspase-8 and pro-caspase-9 and activated both caspase-8 and caspase-9 (Figure 3). All these findings implicated both the mitochondrial pathway and the death receptor pathway in Silibinin-driven apoptosis in AsPC-1, Panc-1, and BxPC-3 cells.

### 2.4. Silibinin Induces Cell-Cycle Arrest in Human AsPC-1, but not in Panc-1 and BxPC-3 Cells

To gain an insight into the mechanism of the anti-proliferative activity of Silibinin, its effects on cell cycle distribution were determined. In general, the progression of cell cycle in eukaryotes is a complex process involving resting G0 phase, and cell growth involving G1, S and G2/M phases in a step-wise manner [17]. Silibinin caused cell cycle arrest in different phases in different cancer cells. For example, Silibinin caused G1 and G2/M cell cycle arrest via distinct circuitries in human prostate cancer PC3 cells [18]. Silibinin inhibits human nonsmall cell lung cancer cell growth through cell-cycle arrest in G1 phase by modulating expression and function of key cell-cycle regulators [19]. Silibinin promotes cell-cycle arrest in G2/M phase in Fet and Geo cell lines and G1 arrest in HCT116 of human colon cancer [17]. In this text, our findings showing that Silibinin induced G1 arrested in AsPC-1 cells *versus* no obvious G1, S or G2 arrest in BxPC-3 and Panc-1 cells under identical treatment conditions, further showed some selectivity and specificity in its biological responses in different pancreatic cancer cell types. It was also important to identify here that Silibinin showed better efficacy in AsPC-1 cells *versus* BxPC-3 and Panc-1 cells following treatments at different doses. All these findings implicated that Silibinin inhibited different pancreatic cancer cells in a different manner. More studies, however, are needed in the future to find the mechanism of Silibinin action. The data presented in this study also supported and warranted Silibinin efficacy studies in pre-clinical pancreatic AsPC-1 models.

## 3. Experimental Section

### 3.1. Cell Line and Reagents

Human pancreatic cancer cells, AsPC-1, Panc-1 and BxPc-3, were purchased from the Chinese Academy of Sciences Cell Bank. Cells were cultured in DMEM medium containing 10% fetal bovine serum (Gibico, NY, USA) and 1% penicillin-streptomycin under standard culture conditions (37 °C, in 95% humidified air containing 5% CO_2_). Silibinin was obtained from Sigma and dissolved in DMSO at concentration of 500 mM.

Annexin-V-FITC Apoptosis Detection Kit I was obtained from BD, PVDF membrane was purchased from Millipore, 3-(4,5-dimethylthiazol-2-yl)-2,5-diphenyltetrazolium bromide (MTT), 4,6-diamidino-2-phenylindile (DAPI), propidium iodide (PI) were purchased from Sigma, Caspase activity assay kits were obtained from Bestbio., DMEM was purchased from Invitrogen Corp. and fetal bovine serum (FBS) was purchased from GIBCO–BRL. Antibodies against Pro-caspase-3, Pro-caspase-8, Pro-caspase-9 and HRP-labeled goat anti-mouse IgG were purchased from Santa Cruz Biotechnology; Antibody against β-actin was obtained from Sigma. All other chemicals not specifically cited here were purchased from Sigma (St. Louis, MO, USA).

### 3.2. Cell Proliferation Assay

To compare the sensitivities of different pancreatic cancer cells to Silibinin treatment, MTT proliferation assays were performed to determine cell viability [19]. Cells (5 × 10^3^) were seeded in 96-well plates, and after 24 h, fed with fresh medium or different doses Silibinin (12.5, 25, 50, 100, 200, 400, and 800 μM) in complete medium. After 24, 48, and 72 h of treatment, 20 μL MTT reagent (5 mg/mL, Sigma) was added to each well for 4 h incubation in a CO_2_ incubator. Thereafter, the medium containing MTT was removed and 150 μL dimethylsulfoxide (DMSO) was loaded into each well to dissolve Formazan crystals. Metabolically active cells were spectrophotometrically quantified at 490 nm by detection of reduced yellow tetrazolium MTT to an intracellular purple formazan. The process was repeated in triplicate for all treatment concentrations to confirm accuracy.

### 3.3. Nuclear Staining with DAPI

After treated with the inhibitor Silibinin, the cells in a 24-well plate were washed with ice-cold phosphate-buffered saline (PBS) and fixed with 70% ice-cold ethanol for 5 min at 4 °C. The fixed cells were then washed with PBS, and stained with 300 μL of DAPI (2.5 μg/mL) solution for 5 min at room temperature in the dark. The nuclear morphology of the cells was then examined by a fluorescent microscope.

### 3.4. Flow Cytometric Analysis: Cell Cycle and Apoptosis

Cell cycle arrest and apoptosis were studied by flow cytometry as described [20,21]. AsPC-1, Panc-1 and BxPc-3 cells were plated in 6-well plate under standard culture conditions. After 24 h, cells were fed with fresh medium and treated with DMSO alone or different doses of Silibinin (μM). After 24, 48, and 72 h of treatment, medium was aspirated, cells were quickly washed twice with ice-cold PBS and trypsinized, and cell pellets were collected. For cell cycle analysis studies, cells were fixed in ice-cold 70% ethanol and then stored at 4 °C overnight. Prior to analysis, the cells were washed twice with PBS, suspended in 0.5 mL of cold PI solution containing 10 μL RNase A (25 μg/mL), 10 μL PI (50 μg/mL), and then incubated at 37 °C for an additional 30 min in the dark. For apoptosis analysis, cells (10^6^) were washed with PBS (0.01 M, pH 7.4) and then resuspended in binding buffer according to the manufacturer’s protocols. Aliquots of the cells were then incubated with Annexin-V- FITC and PI, mixed, and incubated for 15 min at room temperature in the dark. Next, cell cycle distribution and induction of apoptosis were determined by analyzing 15,000 ungated cells using a FACScan cytometer and Cell Quest software (FACSCalibur; Becton-Dickinson, San Jose, CA, USA). All experiments were performed in triplicate.

### 3.5. Caspase Activity Assay

Caspase activities were determined by colorimetric assays using caspase-3, caspase-8 and caspase-9 activation kits according to the manufacturer’s protocols. Briefly, at the end of treatment with Silibinin for 24 h, the cells were washed twice with PBS (0.01 M, pH 7.4) and scraped with a rubber policeman. Cells were centrifuged at 10,000× g at 4 °C, and the cell pellet was lysed in 100 μL of the supplied lysis buffer. The resulting suspension was centrifuged at 10,000× g, 4 °C, 10 min, and 10–20 μL supernatant was used for caspase assay. The supernatants were then collected and incubated at 37 °C, with the supplied reaction buffer, which contained dithiothreitol and substrates. The caspase activity was then determined by measuring changes in the absorbance at 405 nm using a microplate reader.

### 3.6. Western Blotting Analysis

The protocol for Western blotting has been described earlier [22]. Cells were treated with Silibinin and washed twice with ice cold PBS, and gently lysed for 30 min in ice-cold cell lysis buffer (50 mmol/L Tris (pH 8.0), 150 mmol/L NaCl, 0.1% SDS, 1% NP40 and 0.5% sodium deoxycholate) containing proteinase inhibitors (1% Cocktail and 1 mmol/L PMSF). Lysates were centrifuged at 10,000× g for 10 min at 4 °C. Supernatants were collected, and protein concentrations were determined using Bradford assay. For Western blotting analysis, an equal amount of protein was subjected to electrophoresis on an SDS-polyacrylamide gel and transferred to a PVDF membrane by electro blotting. The blots were blocked in Tris buffer saline (TBS) containing 5% non-fat milk and 0.1% Tween-20 (blotting grade) for 1 h, and then probed with the desired antibodies overnight at 4 °C. Membranes were subsequently incubated with appropriate horseradish peroxidase-conjugated secondary antibody for 2 h, and visualized by Western blotting detection reagents containing PCA and luminol.

### 3.7. Statistical Analysis

Statistical analysis was performed with statistical program for social sciences software (SPSS, Chicago, IL, USA) as needed. All data were expressed as mean ± SEM and a statistically significant difference was considered to be present at *p* < 0.05. Unless mentioned otherwise, all the assays were repeated in triplicate in three independent experiments.

## 4. Conclusions

Silibinin treatment of AsPC-1, BxPC-3 and Panc-1 cells resulted in a significant dose- and time-dependent growth inhibition and apoptosis. Silibinin activated caspase 3/8/9 causing apoptotic death of human pancreatic cells and induced a decrease in S phase and cell cycle arrest in G1 phase in AsPC-1 cells, but resulted in no changes in BxPC-3 and Panc-1 cells. Silibinin showed some selectivity and specificity in biological responses in different pancreatic cancer cell types and better efficacy in AsPC-1 cells *versus* BxPC-3 and Panc-1 cells. Therefore, Silibinin may circumvent the resistance mechanisms that limit the success of conventional drugs, providing a strong rationale for future studies evaluating preventive and/or intervention strategies for Silibinin in pancreatic cancer pre-clinical models.

## Figures and Tables

**Figure 4.**
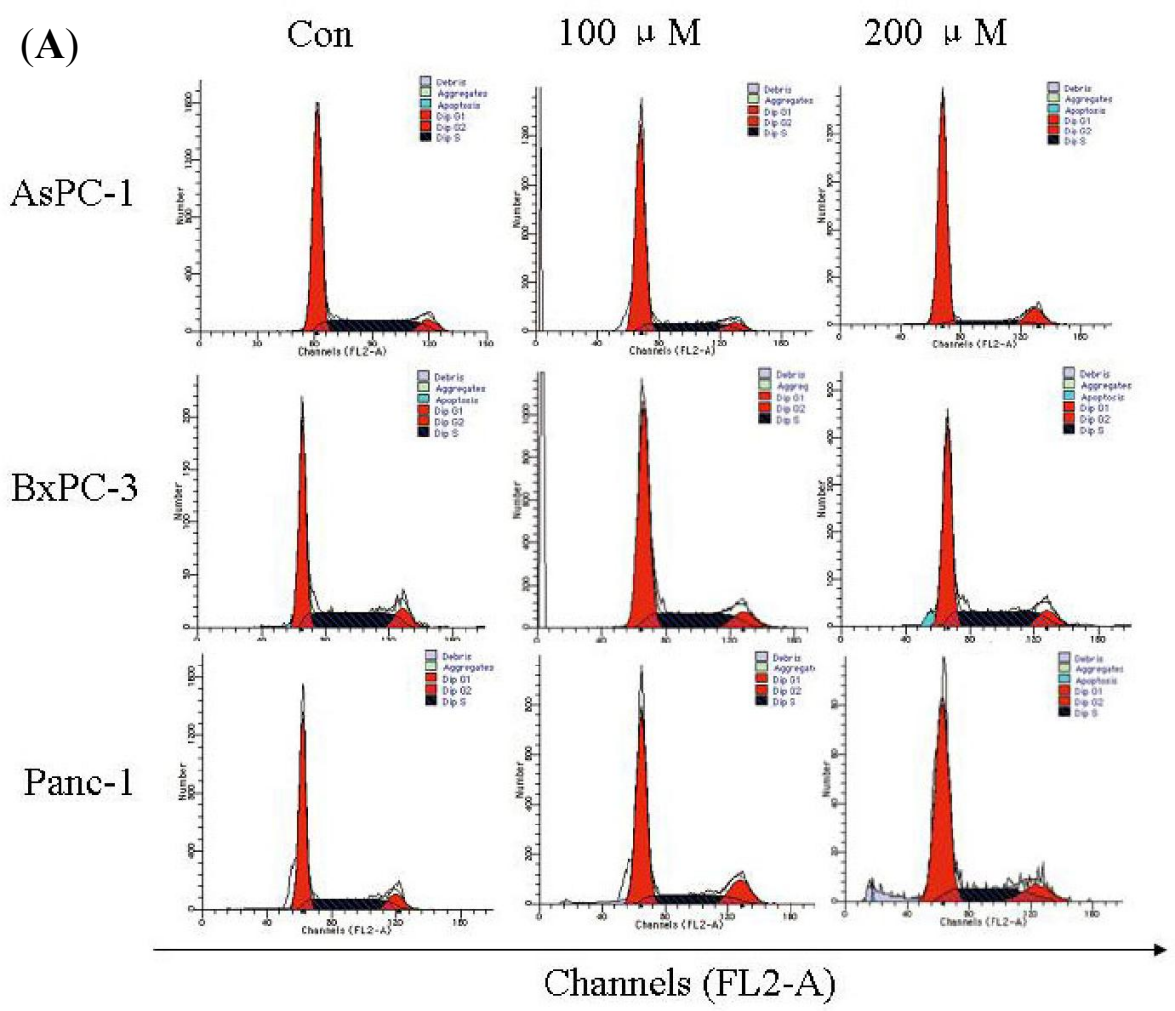

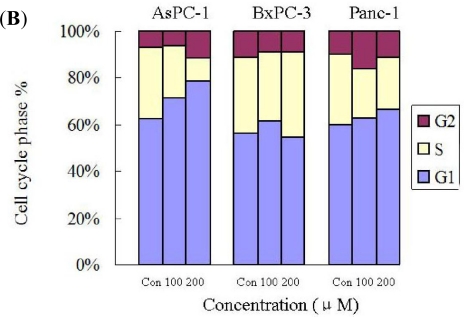
Silibinin triggers cell cycle arrest. (**A**) The DNA content of cells treated with Silibinin for the indicated times was analyzed by flow cytometry. Cell-cycle distributions after treatment with 100, 200 μM Silibinin for 24 h. DMSO as the negative control. Cell-cycle distributions were assessed by PI staining. The DNA content of 15,000 events was analyzed by flow cytometry; (**B**) The number of G1, S and G2 phase cells from Figure 4A.
